# Effects of Compositional Tailoring on Drug Delivery Behaviours of Silica Xerogel/Polymer Core-shell Composite Nanoparticles

**DOI:** 10.1038/s41598-018-31070-9

**Published:** 2018-08-29

**Authors:** Wenfei Huang, Chi Pong Tsui, Chak Yin Tang, Linxia Gu

**Affiliations:** 1 Department of Industrial and Systems Engineering, The Hong Kong Polytechnic University, Hung Hom, Kowloon, Hong Kong China; 2 Department of Mechanical and Materials Engineering, University of Nebraska-Lincoln, Lincoln, NE 68588-0656 USA

## Abstract

Conventional core-shell polymer nanoparticles usually exhibit a rapid release rate with their release kinetics mainly adjusted through changing composition of the polymer shells, limiting their applications for prolonged drug delivery. As a solution to these problems, silica xerogel/polymer core-shell-structured composite nanoparticles have been proposed. Different with our previous work centring on studying process variables, we here focused on investigating the effects of key compositional variables on essential properties of the composite nanoparticles. The drug release profiles (*in vitro*) were well interpreted by the Baker and Lonsdale model on a predicted two-stage basis. The first stage (<1 day) was well controlled from 18.6% to 45.9%; the second stage (1–14 days) was tailored in a range from 28.7 to 58.2% by changing the composition of the silica xerogel cores and polymeric shells. A substantial achievement was reducing the release rate by more than 40 times compared with that of conventional polymer nanoparticles by virtue of the silica xerogel cores. A semi-empirical model was also established in the first attempt to describe the effects of polymer concentration and drug loading capacity on the size of the composite nanoparticles. All these results indicated that the composite nanoparticles are promising candidates for prolonged drug delivery applications.

## Introduction

The demands for developing effective drug delivery systems will continue to expand substantially in the foreseeable future because of increasing numbers of patients and medical treatments. The characteristics of controlled drug delivery systems, in terms of high therapeutic and low toxic effects, have attracted considerable attention for decades^1,2^. Biodegradable polymers such as poly(lactic acid) (PLA), poly(glycolic acid) (PGA), and poly(lactic-*co*-glycolic acid) (PLGA) have been widely adopted as the primary components of controlled drug delivery systems because of their biocompatibility and controllable biodegradability^3–5^. Various active ingredients, including proteins, antibiotics, anti-cancer drugs, and DNA, have been successfully encapsulated by these biomaterials as core-shell polymer nanoparticles^6–8^.

For these conventional core-shell nanoparticles, drug release is characterized by the penetration of hydrophilic active molecules through the polymeric shells. Therefore, the drug release profiles usually follow the degradation kinetics of the biodegradable polymers. The drug release kinetics of these polymer nanoparticles, PLGA for instance, are usually adjusted by changing the composition of the polymeric shells in terms of the lactide/glycolide ratio and molecular weight (MW)^6,9^. Regardless of these compositional adjustments, the drug release of polymer nanoparticles usually ranges from several hours to several days when hydrophilic small drug molecules (e.g., vancomycin, an antibiotic) are encapsulated^10–13^. Vancomycin-loaded PLGA core-shell nanoparticles have been found to reach complete release within 24 h^14^. For treating chronic diseases such as diabetes, long-term drug delivery, in the range from weeks to months or even longer, is important for patient compliance^1,15,16^. The relatively fast release rates of polymer nanoparticles are not suitable for long-term drug delivery applications. The exploration of an alternative or additional route to prolong the release and control the release kinetics is therefore necessary.

A double emulsion/solvent evaporation technique is usually used for the preparation of core-shell polymer nanoparticles^13,14,17^. However, hydrophilic actives tend to leak into the external aqueous phase in the second emulsion, which leads to an undesirable waste of actives and, thus, to low encapsulation efficiency^8,18,19^. This phenomenon is attributed to the emulsification energy tending to break the polymer shell and accelerate the diffusion of hydrophilic actives into the aqueous phase^19–21^. This problem is even more severe for the encapsulation of hydrophilic small molecules such as antibiotics. Unlike hydrophilic macromolecules (such as proteins), hydrophilic small molecules are more likely to diffuse into the aqueous phase through the hydrophobic polymer matrices^8,22^. One strategy to address this issue is to exploit the ionic interactions between charged polymers and active molecules. However, this route is only applicable to charged molecules and its applications are greatly limited by the choice of polymers and active molecules^23^. Another strategy is to use additives to stabilize the core materials (i.e., the internal phase containing the active molecules) of the core-shell-structured nanoparticles. Deepak *et al*. has successfully enhanced the encapsulation efficiency (40.5%) of gemcitabine HCl by stabilizing the core materials of core-shell PLGA nanoparticles by initiating the crosslinking of BSA in the core materials^8^. Yang *et al*. improved the encapsulation efficiency of protein and peptide from 32% to 57% and from 22% to 62%, respectively, in core-shell lipid nanoparticles by incorporating hydrogel cores (e.g., Pluronic gel and glyceryl palmitostearate)^24^. Compared with the strategy of ionic interaction, the route of core stabilization is more advantageous because the choices of active molecules and polymers are less restricted. Even though the encapsulation efficiency in the aforementioned cases were enhanced to a great extent, the drug release in these systems is accomplished within only several days, which is still too fast to be applicable for long-term release^25–27^. Moreover, large gaps exist between encapsulation efficiencies of these systems and complete encapsulation, indicating potential opportunities for further improvement.

To fill these gaps, we introduced silica xerogel as a core stabilizer to prevent the leakage of encapsulated drugs in a previous study^28^. Silica xerogel, serving as an amorphous, bioresorbable and biocompatible sol-gel-derived SiO_2_ matrix, is a promising biomaterial for drug delivery^26,29–31^. The proposed composite nanoparticles were designed to enhance encapsulation efficiency via the adjustment of process variables and better manipulate drug release behaviour via the adjustment of compositional variables. To fabricate these composite nanoparticles, we developed a novel gelation-emulsion method in our previous work with a focus on enhancing the encapsulation efficiency and adjusting the particle size through controlling key process variables^28,32^. The average diameters of the composite nanoparticles were adjusted in the range from 192 to 569 nm, with a maximum encapsulation efficiency as high as 82.2% by adjusting the process variables^28^. On the basis of these preliminary results, we deduced the optimal combination of process variables, which paved the way for the current study of the effects of compositional variables on controlled drug release. The most important function of the silica xerogel core was to better control the release kinetics and prolong drug release, which was not covered in our previous work. The drug release from the silica xerogel is governed by the physical properties of the xerogel, such as its porosity, density and internal structure, which are easily controlled by adjusting the parameters of the sol-gel chemistry^33–35^.

In the current study, we focused on investigating the effects of compositional parameters in controlling the drug release kinetics, size and encapsulation efficiency of drug-loaded core-shell silica xerogel/polymer composite nanoparticles. The composite nanoparticles loaded with a model drug, vancomycin, were fabricated using the gelation emulsion method. PLGA and poly((d,l-lactic acid-*co*-glycolic acid)-*block*-ethylene glycol) (PLGA-PEG) were selected as the components of the polymer shell. Control of the drug release kinetics of the fabricated composite nanoparticles was achieved by tailoring the structure and composition of the silica xerogel core and polymeric shell. The unique release kinetics of the composite nanoparticles were well elucidated via mathematical kinetic models. The influence of compositional factors (i.e., polymer concentration, drug loading, type of PLGA-PEG and gelation behaviour of the silica xerogel) on the encapsulation efficiency and particle size of the composite nanoparticles were also investigated theoretically and experimentally. These fabricated composite nanoparticles are promising candidates for drug delivery applications where a larger particle size is preferred, such as drug delivery to macrophages^36^.

## Results and Discussion

### Characterization of composite nanoparticles

**Figure 1:**
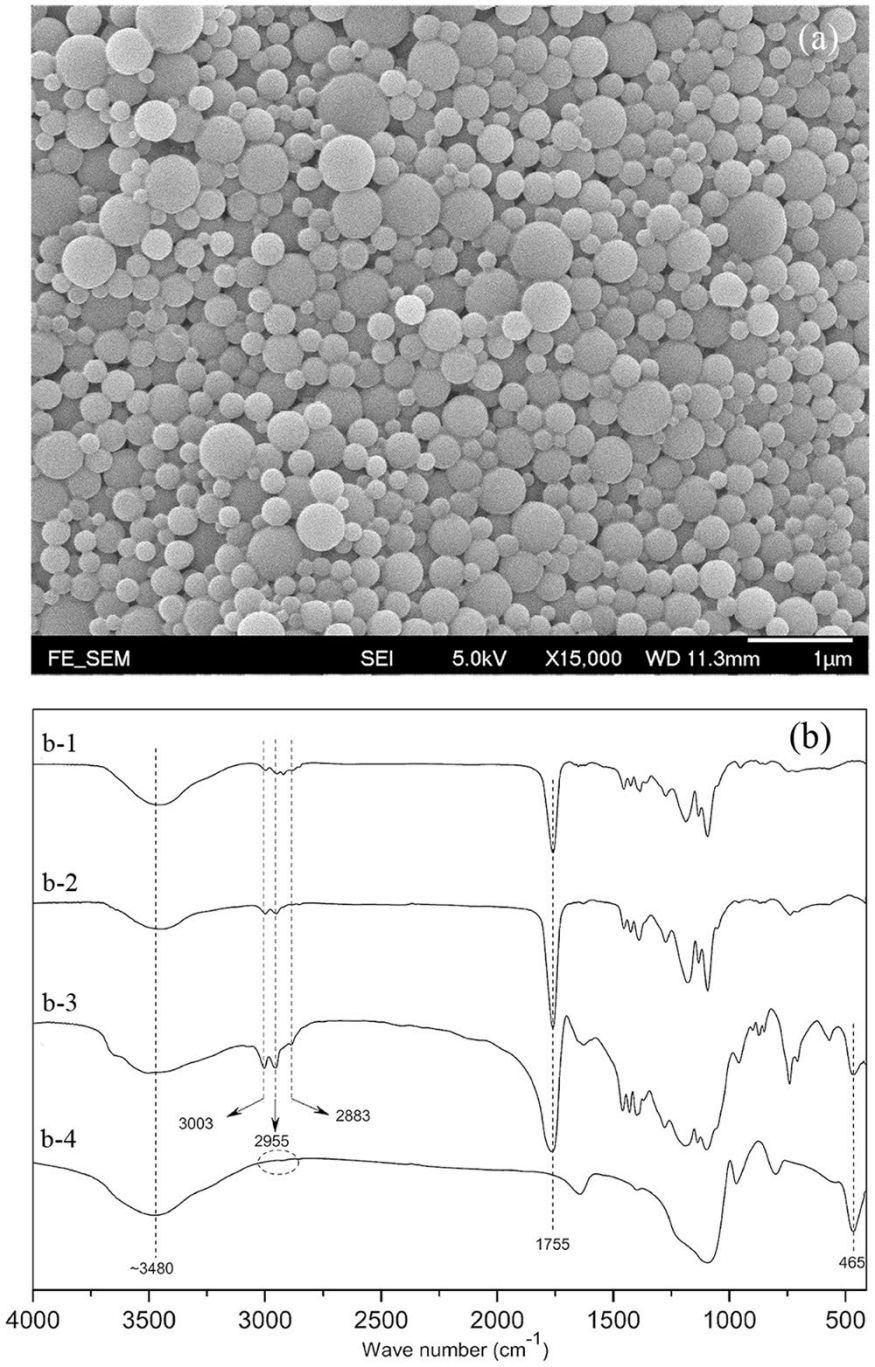
(**a**) SEM image of composite nanoparticles; (**b**) FT-IR spectra of (b-1) PEG-PLGA, (b-2) PLGA, (b-3) composite nanoparticle and (b-4) silica xerogel.

The prepared vancomycin-loaded silica xerogel/polymer composite nanoparticles were spherical and smooth, as shown in Fig. 1(a). The particle size was tailored in the range from 230 to 925 nm by adjusting the compositional parameters of the composite nanoparticles, as discussed in the following section. The sample appeared in Fig. 1(a) was prepared with the parameters of polymer concentration: 30 mg/ml, sonication time: 1.5 minutes and the ratio of water/tetraethyl orthosilicate (*R*_*W/T*_). The particle size distribution of the sample with a mean size of 301.8 nm in diameter was shown in Fig. S1.

The Fourier Transform Infrared (FT-IR) spectra of the PLGA, PLGA-PEG, composite nanoparticles and the silica xerogel are illustrated in Fig. 1(b). The sharp peak at 1755 cm^−1^ corresponds to the stretching vibration of the ester C=O bonds of PLGA and PLGA-PEG. The small peaks at 3003 cm^−1^ and 2955 cm^−1^ are assigned to the symmetric stretching vibration of the C−H bonds of the −CH_3_ groups of the PLGA and PEG-PLGA, respectively. The peak at 2883 cm^−1^ is ascribed to the C−H stretching vibration of −CH_2_. The absence of the characteristic peaks of the −CH_3_ and −CH_2_ groups in the spectrum of the silica xerogel indicates that all of the tetraethyl orthosilicate (TEOS) was hydrolysed. The bending vibrations of Si−O−Si bonds are characterized by the peak at 465 cm^−1^, which confirms the successful incorporation of the silica xerogel into the composite nanoparticles. The broad peaks at approximately 3480 cm^−1^ result from the −OH stretching vibrations of the PLGA, PLGA-PEG and the silica xerogel.

### Study of PEG-PLGA

To extend the circulation time of the drug delivery vesicles, PEG groups should be incorporated into the surface of the nanoparticles. Ensuring that gelation occurs in the silica solution nanodroplets (also known as nanoreactors) without significant agglomeration requires a biocompatible emulsion stabilizer. To address both of the aforementioned targets simultaneously, PEG-PLGA was blended with PLGA to compose the polymeric shells. As an emulsifier, a proper hydrophilic-lipophilic balance (HLB) value of PEG-PLGA was necessary to stabilize the primary emulsion (W/O emulsion)^37,38^.

**Figure 2:**
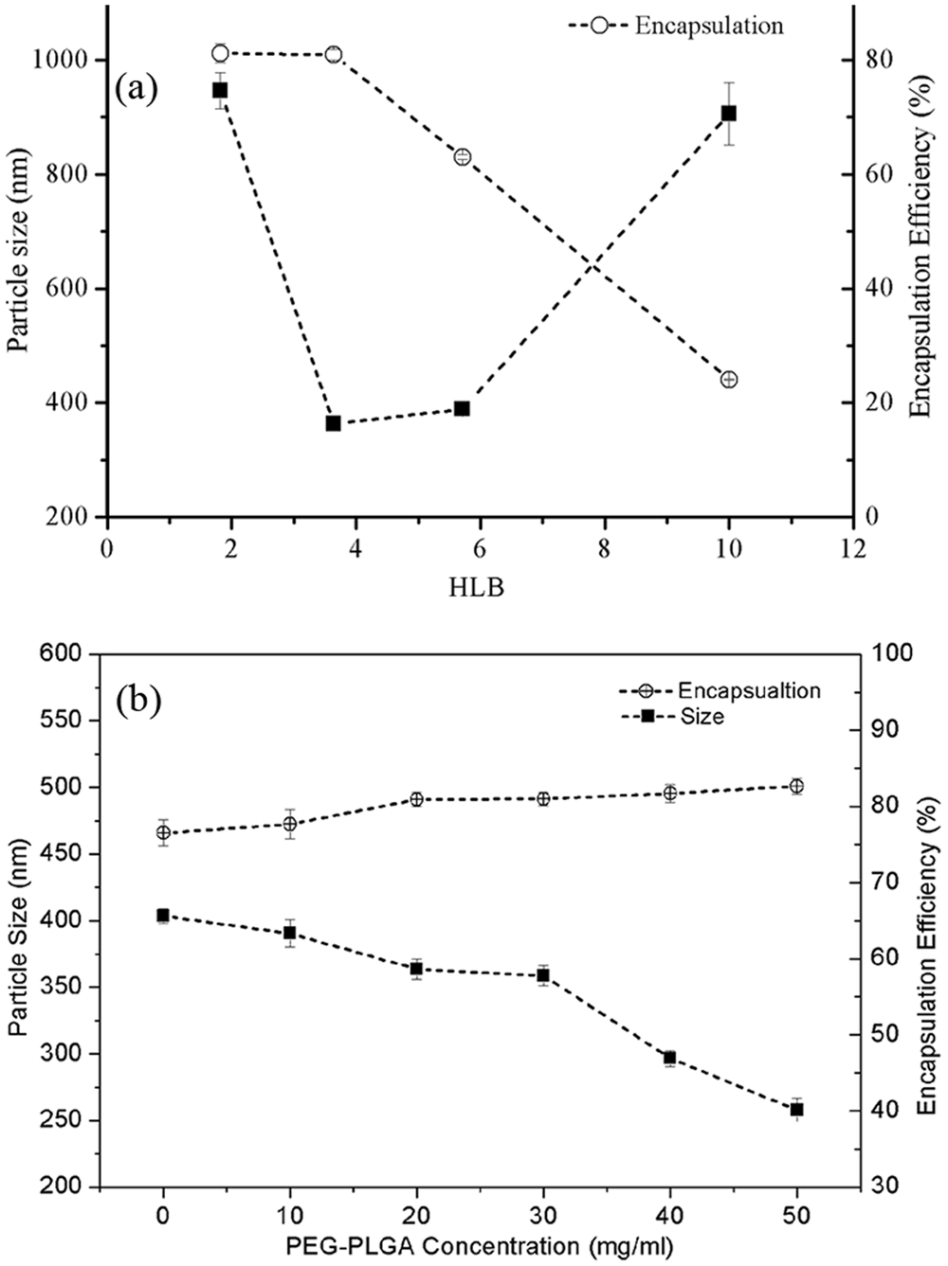
Effects of (**a**) different HLB values and (**b**) concentrations of PEG-PLGA on mean size and encapsulation efficiency of the composite nanoparticles (Fabrication parameters: total polymer concentration = 50 mg/ml; sonication time = 1.5 minutes; R_W/T_ = 40; Drug loading = 3%).

To design the proper structure of PEG-PLGA with an optimal HLB value, PEG-PLGA with different HLB values was prepared for the fabrication of the composite nanoparticles. Figure 2(a) shows that a small particle size and high encapsulation efficiency can be attained with a stable emulsion in our fabrication process. Moreover, the optimal emulsion stability was achieved with an HLB value of 3.64. With this optimal HLB value, the influences of the PEG-PLGA concentration on the size and encapsulation efficiency were investigated; the results, shown in Fig. 2(b), indicate that an increase in the PEG-PLGA content enhanced the drug encapsulation efficiency slightly and reduced the size (mean diameter) of the composite nanoparticles prepared with a constant polymer concentration. This result is attributed to a higher content of PEG-PLGA as an emulsifier stabilizing the primary emulsion. In addition, the prepared composite nanoparticles exhibited strong adhesive forces and tended to agglomerate at room temperature when the weight ratio of PEG-PLGA to PLGA was greater than 2:3. For this reason, the optimal composition of the polymer shell was deduced to be a weight ratio of 2:3 (PEG-PLGA: PLGA).

### Study of polymer concentration

A theoretical analysis was first performed to investigate the effects of the polymer concentration on the size of the composite nanoparticles. Under a constant volume of the organic phase, the polymer concentration was directly linked to the added polymer. Assuming that the composite nanoparticles composed polymeric shells and silica-based cores (containing silica solution and drug molecules), the entire volume (*V*_*p*_) of an individual core-shell structure composite nanoparticle before being completely dried is expressed as:

1 $${V}_{p}={V}_{s}+{V}_{d}+{V}_{si}$$

where *V*_*s*_, *V*_*si*_ and *V*_*d*_ are the volumes of the polymer shell, the silica solution and the loaded drugs of an individual composite nanoparticle, respectively. Assuming that all the added silica solution and polymer solution are transformed into cores and shells of the composite nanoparticles without agglomeration in each batch of fabrication, the relationship described by Equation (1) can be written in the form

2 $${V}_{p}=\frac{{C}_{sol}{V}_{sol}}{N{\rho }_{s}}+\frac{mE}{N{\rho }_{d}}+\frac{{V^{\prime} }_{si}}{N}$$

where *C*_*sol*_, *V*_*sol*_, *ρ*_*s*_, *ρ*_*d*_, *m, E* and ${V^{\prime} }_{si}$ are the polymer concentration, the volume of the added polymer solution in each batch, the density of the solid polymeric shell, the density of the incorporated drugs, the weight of the incorporated drug in each batch, the encapsulation efficiency and the volume of added silica solution, respectively; *N* is the total number of fabricated composite nanoparticles in a single batch (refer to the fabrication process in “Methods”).

The diameter (*d*_*p*_) of the composite nanoparticle can then be expressed as

3 $${{d}_{p}}^{3}=\frac{6{C}_{sol}{V}_{sol}}{\pi N{\rho }_{s}}+\frac{6mE}{\pi N{\rho }_{d}}+\frac{6{V^{\prime} }_{si}}{N\pi }$$

Equation (3) was developed on the basis of an ideal situation in which each of the prepared composite nanoparticles possesses the same diameter; however, this assumption is not always valid. In a real case, the sizes of the composite nanoparticles are not all the same and usually exhibit a distribution of values. To accommodate these variations, empirical coefficients were introduced to calibrate Equation (3). Formation of the emulsions largely depends on process parameters such as emulsification power and time. As all the process parameters of the emulsification were fixed during the study of compositional factors, the number (*N*) of the prepared composite nanoparticles was assumed to be constant in the present study. This number was estimated empirically because of the lack of an effective method to calculate the number of nanoparticles. Considering the aforementioned situations, the mean diameter of the prepared composite nanoparticles (*d*_*av*_) can be expressed as

4 $${{d}_{av}}^{3}={B}_{1}\frac{{C}_{sol}{V}_{sol}}{{\rho }_{s}}+{B}_{2}\frac{mE}{{\rho }_{d}}+{B}_{3}{V^{\prime} }_{si}$$

where *B*_1_, *B*_2_ and *B*_3_ are the empirical factors that account for variations of both the size distribution and the empirically obtained number (*N*).

To study the effects of polymer concentration, all of the parameters other than the polymer concentration (*C*_*sol*_) were kept constant. The encapsulation efficiency was assumed to be constant, regardless of the slightly difference as shown in Fig. 2(b); this assumption is incorporated into the determination of the empirical factor *B*_2_. In this case, a linear relationship model of the cube mean diameter of composite nanoparticles (*d*_*av*_^3^) and the concentration of the polymer solution (*C*_*sol*_) is obtained as

5 $${{d}_{av}}^{3}={B}_{1}\frac{{V}_{sol}}{{\rho }_{s}}{C}_{sol}+{B}_{2}\frac{m}{{\rho }_{d}}+{B}_{3}{V^{\prime} }_{si}+\varepsilon $$

where *ε* is an error term due to measurement error. Given that the volume of added silica solution (${{V}_{si}}^{\text{'}}$) in each batch was unchanged, Equation (5) can be transformed to

6 $${{d}_{av}}^{3}={B}_{1}\frac{{V}_{sol}}{{\rho }_{s}}{C}_{sol}+{B}_{2}\frac{m}{{\rho }_{d}}+{B}_{0}$$

where *B*_0_ is an empirical factor covering both the error term (*ε*) and ${B}_{3}{V^{\prime} }_{si}$. According to the Equation (6), the cubic mean diameter is linearly related to the polymer concentration. This relationship was experimentally verified; the results are discussed in the following.

On the basis of the definition of drug loading capacity (*C*), the amount of added drug (*m*) in Equation (6) can be expressed as $\,m={C}_{sol}{V}_{sol}C$. Equation (6) can then be transformed into the following form with consideration of the drug loading capacity:

7 $${{d}_{av}}^{3}={B}_{1}\frac{{C}_{sol}{V}_{sol}}{{\rho }_{s}}+{B}_{2}\frac{{C}_{sol}{V}_{sol}}{{\rho }_{d}}C+{B}_{0}$$

In the study of the effects of drug loading, all the factors were kept constant except the drug loading capacity (*C*). A linear relationship between the cubic average diameter of the composite nanoparticles (*d*_*av*_^3^) and the drug loading capacity (*C*) is observed in Equation (7) and was verified by the experimental results.

**Figure 3:**
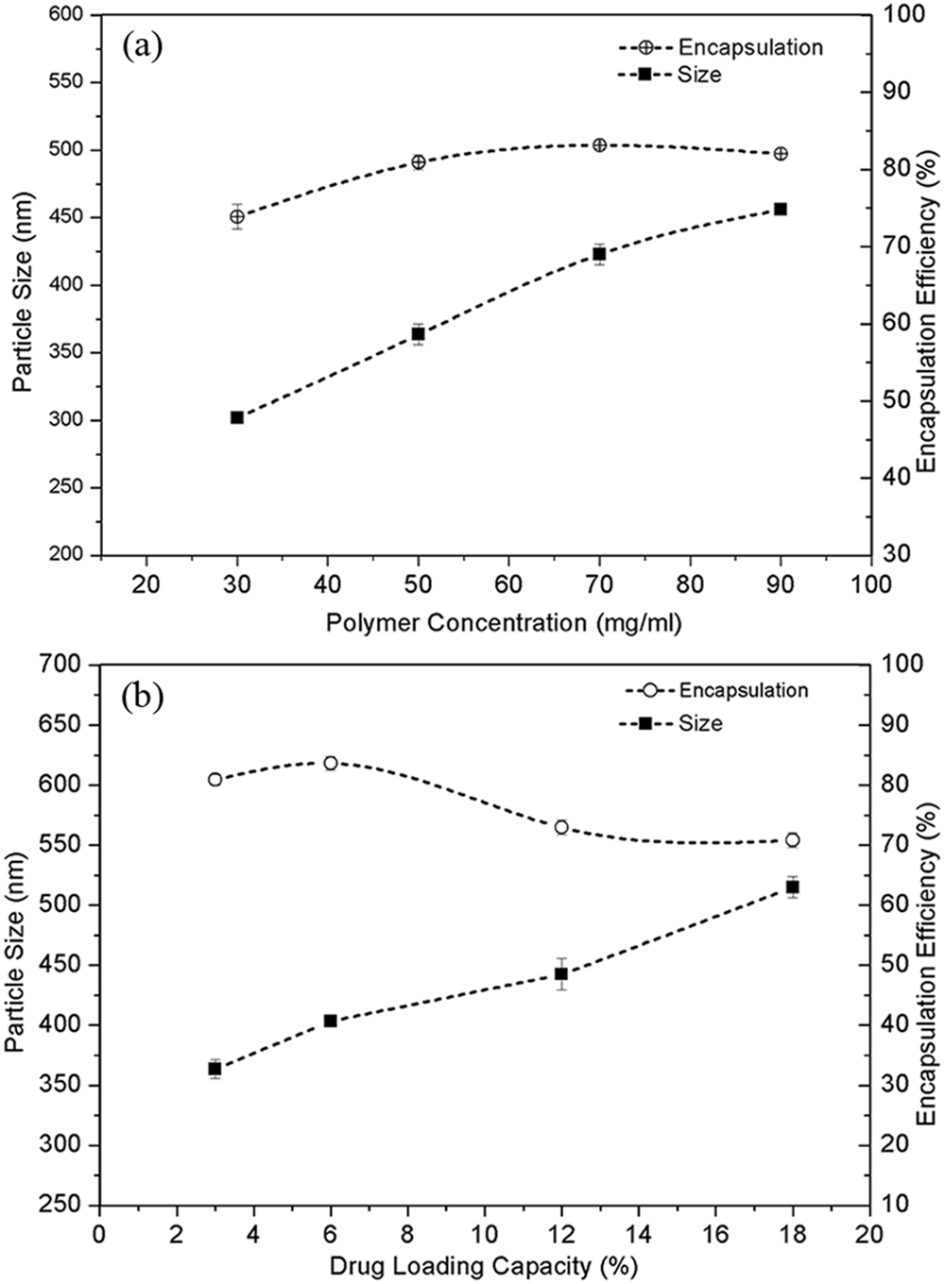
Effects of (**a**) the polymer concentration and (**b**) the drug loading capacity on mean size and encapsulation efficiency of composite nanoparticles (fabrication parameters: PEG-PLGA/PLGA ratio = 2:3; sonication time = 1.5 minutes; *R*_*W/T*_ = 40).

Figure 3(a) shows that the particle size and the encapsulation efficiency increase with increasing total polymer concentration at the optimal HLB value and the optimal concentration of PLGA-PEG. The addition of more polymer resulted in an increase in the viscosity of the polymer solution, which contributed to the formation of larger particles in the second emulsion under the same emulsification energy. The likelihood of particle breakage was much less in a more viscous polymer solution under the same emulsification energy, explaining the observation that the drug encapsulations were higher at higher polymer concentrations. The encapsulation efficiency increased substantially when the polymer concentration was increased from 30 mg/ml to 50 mg/ml. A higher encapsulation efficiency indicates better drug encapsulation, which also influences drug release from the composite nanoparticles. This influence is discussed in the section ‘Modelling of drug release kinetics’.

A linear relationship between the cubic diameter of the composite nanoparticles (*d*_*p*_^3^) and the polymer concentration (*C*_*sol*_) is observed (Fig. S2(a)). The *R*-squared value was 0.99, confirming the linear relationship between *C*_*sol*_ and *d*_*p*_^3^ as described in Equation (6). In addition, the slope coefficient of the regression was sufficiently significant to express the linear relationship (Table S1; a significance level smaller than 0.10 was considered to be acceptable in this study). On the basis of these regression results (Table S1), the empirical factor *B*_1_ of Equation (6) was determined to be 15.42 × 10^4^ by solving the following equation:

$${B}_{1}\frac{{V}_{sol}}{{\rho }_{s}}=1.15\times {10}^{6}({\rm{slope}}\,{\rm{coefficient}})$$

(*ρ*_*s*_ = 1.34 g/ml, *V*_*sol*_ = 10 ml). The intercept of the regression was then used to calculate the empirical factor *B*_0_ in the following discussion after the value of *B*_2_ was obtained.

### Study of drug loading capacity

A high drug loading capacity of a drug delivery system is always preferred because it can minimize the use of polymers. From Fig. 3(b), increasing the drug loading capacity of the composite nanoparticles enhances the encapsulation efficiency to reach 84% at a loading capacity of 6%. An increase in size is also observed along with the increasing drug loading capacity because a higher drug amount led to a larger volume of the cores and, thus, to larger average sizes of the composite nanoparticles.

The relationship between the drug loading capacity (*C*) and the cubic diameter (*d*_*p*_^3^) of the composite nanoparticles is illustrated in Fig. S2(b). A high *R*-squared value of 0.97 was also obtained, confirming the effectiveness of Equation (7) in determining the effect of drug loading capacity on the particle size. On the basis of the results in Table S2, *B*_2_ was calculated to be 18.67 × 10^3^ by solving the following equation:

$${B}_{2}\frac{{C}_{sol}{V}_{sol}}{{\rho }_{d}}=5.66\times {10}^{6}({\rm{slope}}\,{\rm{coefficient}})$$

(*ρ*_*d*_ = 1.65 g/ml*, V*_*sol*_ = 10 ml, *C*_*sol*_ = 50 mg/ml). Because the error terms of two regressions for calculating the values of B_1_ and B_2_ are different, there are two values for B_0_. After acquiring the values of *B*_1_ and *B*_2_, the two values of *B*_0_ were obtained by applying the intercepts from the two regression results. One *B*_0_ value was determined to be −7.78 × 10^6^ by solving the following equation using the intercept of regression from Table S1: ${B}_{2}\frac{m}{{\rho }_{d}}+{B}_{0}=-\,7.61\times {10}^{6}$(*m* = 15 mg, *ρ*_*d*_ = 1.65 g/ml, *B*_2_ = 9.77 × 10^3^). The other *B*_0_ value was calculated to be −28.5 × 10^6^ by solving the following equation using the intercept of regression from Table S2: ${B}_{1}\frac{{V}_{sol}}{{\rho }_{s}}{C}_{sol}+{B}_{0}=29.00\times {10}^{6}$(*V*_*sol*_ = 10 ml, *C*_*sol*_ = 50 mg/ml*, ρ*_*s*_ = 1.34 g/ml, *B*_1_ = 8.07 × 10^4^). In this case, *B*_0_ varies from −28.5 × 10^6^ to −7.78 × 10^6^ under the current conditions.

The following semi-empirical model describing the relationship of the average diameter and polymer concentration/drug loading capacity was obtained:

8 $${{d}_{av}}^{3}={B}_{1}\frac{{V}_{sol}}{{\rho }_{s}}{C}_{sol}+{B}_{2}\frac{{V}_{sol}}{{\rho }_{d}}{C}_{sol}C+{B}_{0}$$

(*B*_1_ = 15.42 × 10^4^, *B*_2_ = 18.67 × 10^3^, −28.5 × 10^6^ ≤ *B*_0_ ≤ −7.78 × 10^6^, *V*_*sol*_ = 10 ml, *ρ*_*d*_ = 1.65 g/ml, *ρ*_*s*_ = 1.34 g/ml, 10 ≤ *C*_*sol*_ ≤ 90 mg/ml, 3 ≤ *C* ≤ 18%).

### Study of gelation behaviours

The gelation behaviours of the silica solution, which functions as a core stabilizer, influence the size and encapsulation efficiency of the prepared composite nanoparticles. The gelation or condensation of the silica solution was triggered after the addition of alkali to the hydrolysis solution during the first step. The gelation rate or gelation time is directly linked to the alkali content (NH_3_ solution in the present study)^39^. A low gelation rate causes the leakage of drug molecules during the sonication process because wet gel cannot be completely formed to prevent the escape of the drug molecules to the external aqueous phase. Therefore, a fast gelation rate was preferred in our fabrication.

**Figure 4:**
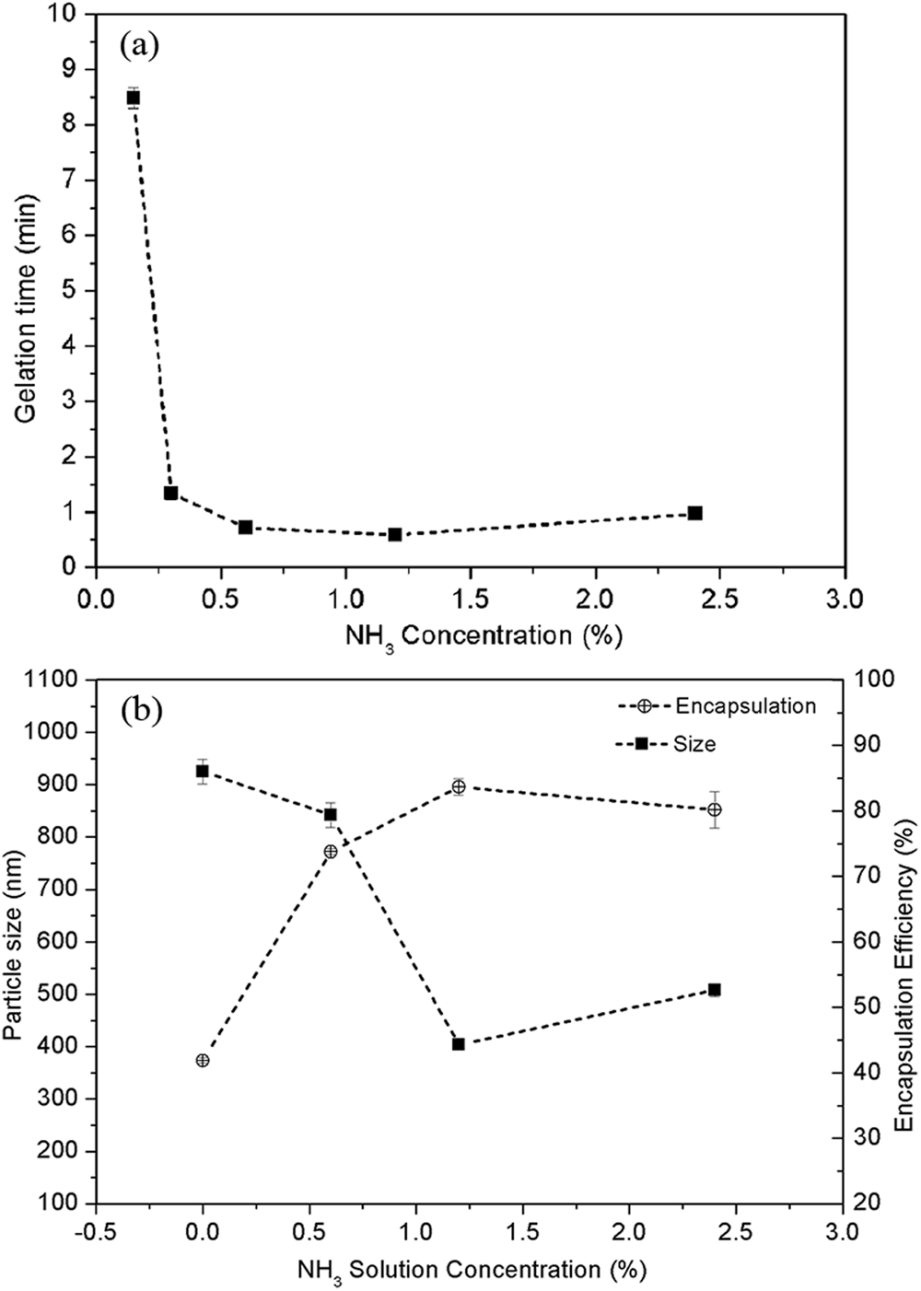
(**a**) Relationships between the required time for the formation of bulk silica wet gel and the added NH_3_ concentrations when R_W/T_ = 20; (**b**) Effect of the concentration of NH_3_ solution on mean size and encapsulation efficiency of composite nanoparticles (fabrication parameters: PEG-PLGA/PLGA ratio = 2:3; polymer concentration = 50 mg/ml; sonication time = 1.5 minutes; *R*_*W/T*_ = 40; drug loading = 6%).

The concentration of the silica solution was expressed as the ratio of water/TEOS (*R*_*W/T*_ ratio) with the opposite trend, such that the highest concentration of silica solution corresponded to the lowest *R*_*W/T*_ ratio. To investigate the gelation rate, the lowest *R*_*W/T*_ ratio (20) was used. As confirmed in Fig. 4(a), the gelation time decreases substantially from 0.15% to 6% NH_3_ concentration and reaches the lowest value at the concentration of 0.6% and 1.2%. Decreasing the NH_3_ concentration leads to an increase in the gelation time. Without the addition of the alkali, the gelation time was longer than two weeks. With increasing alkali content (the NH_3_ concentration in the present study), decreasing gelation times were observed until reaching the lowest point. The lowest water/TEOS (*R*_*W/T*_) ratio was 20 in this study because a lesser amount of drug would dissolve in the silica solution with a lower water content of silica solution (lower *R*_*W/T*_ ratio).

From Fig. 4(b), the decreasing encapsulation efficiency is observed at a low concentration of NH_3_ (R_W/T_ ratio was kept constant at 40), which was caused by the gelation rate being too slow to effectively stabilize the core of the composite nanoparticles and thus results in severe drug leakage during the emulsification. The unreacted silica solution therefore tended to flow out from the particles and deposit onto the surfaces of the composite nanoparticles, leading to their agglomeration. The optimal gelation rate was achieved at an NH_3_ concentration of 1.2% at an *R*_*W/T*_ ratio of 40, as judged by the maximum value of encapsulation efficiency (84%) and the smallest particle size (403 nm).

**Figure 5:**
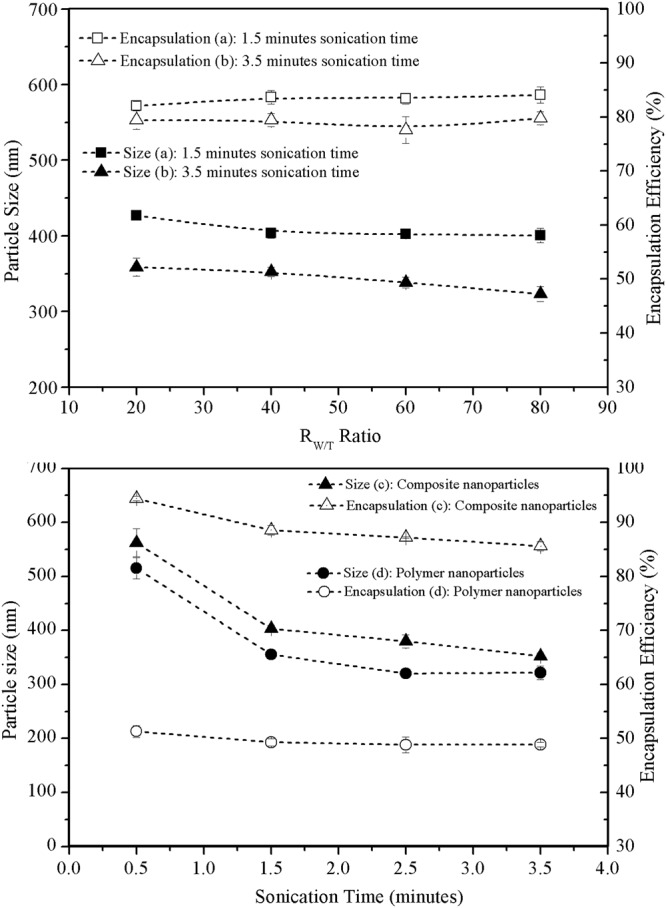
Effects of different *R*_*W/T*_ ratios and sonication time on mean size and encapsulation efficiency of composite nanoparticles and polymer nanoparticles: (**a**) 1.5-minute sonication time; (**b**) 3.5-minute sonication time (fabrication parameters: PEG-PLGA/PLGA ratio = 2:3; polymer concentration = 50 mg/ml; drug loading = 6%; sonication power: 100 W); effects of sonication time of the second emulsion process on mean size and encapsulation efficiency of (**c**) composite nanoparticles and (**d**) polymer nanoparticles (Fabrication parameters: PEG-PLGA/PLGA ratio = 2:3; polymer concentration = 50 mg/ml; *R*_*W/T*_ = 40; Drug loading = 6%; sonication power: 100 W).

The influence of the *R*_*W/T*_ ratio on the encapsulation efficiency and size of the composite nanoparticles was less significant than that of the NH_3_ solution concentration, as shown in Fig. 5(a,b). No significant change in the encapsulation efficiency or particle size is observed at different *R*_*W/T*_ ratios. A similar trend is observed even at a different sonication time. A significant size reduction is observed (at a significance level of 0.05) for the composite nanoparticles fabricated with a longer sonication time. Unlike the NH_3_ solution, the increase of the *R* ratio did not contribute to the deferred gelation process because the environment contained insufficient alkali. Instead, the gelation occurred rapidly after the addition of the NH_3_ solution, as indicated by the constant encapsulation efficiency.

### Comparison with polymer nanoparticles

The size and encapsulation efficiency of the prepared composite nanoparticles and conventional polymer nanoparticles fabricated with different sonication times for the second emulsion are compared in Fig. 5(c,d). Student’s *t*-test with a significance level of 0.05 revealed a statistically significant improvement in the encapsulation efficiency for composite nanoparticles. The encapsulation efficiency of the prepared composite nanoparticles was 2–3 times higher than that of the conventional PLGA nanoparticles, which indicates that the silica xerogel is an effective core stabilizer for improving drug encapsulation. In the absence of the silica xerogel, drug molecules are likely to leak out even under a short emulsification time. Because the drug molecules were trapped in the networks of the silica xerogel, their escape from the nanoparticles was limited even under a greater emulsification intensity.

### Study of drug release kinetics

Nanoparticles with different structures and drug distributions undergo different drug release mechanisms. Because the fabricated composite core-shell nanoparticles were composed of silica xerogel as the primary core materials and biodegradable polymers as the shells, the erosion behaviour of the polymeric shells should be the same as that of conventional polymer nanoparticles. However, the drug molecules dissolved and diffused in the silica xerogel, which was different from conventional polymer nanoparticles. Therefore, the overall drug release behaviours of the composite nanoparticles differ from those of conventional polymer nanoparticles. To interpret the drug release behaviours of the composite nanoparticles, mathematical models with consideration of one or more of the characteristic drug release behaviours of composite nanoparticles were first analysed in terms of the erosion behaviours of biopolymer and the drug release behaviours from the polymeric matrices.

Because the silica xerogel core was encapsulated by the polymeric shell, the drug release may be associated with the degradation behaviours of the biodegradable polymer. Drug release from the biodegradable polymer matrix is usually characterized by the following first-order kinetics equation on the basis of its hydrolytic degradation behaviour^40^:

9 $$\mathrm{ln}(1-\frac{{M}_{t}}{{M}_{\infty }})=-\,kt$$

where *M*_*t*_ and *M*_*∞*_ are the fractions of the released drug at time *t* and at infinite time, respectively, and *k* is the release rate constant.

Drug release from porous sol-gel matrices has been demonstrated to occur primarily through diffusion-controlled mechanisms^26,41,42^. Derived from Fick’s first law of diffusion, the Higuchi square root of time model has been widely adopted to describe the drug release behaviours from the silica xerogel matrices because this model is applicable to homogeneous planar or porous polymers in cases where the matrix is reasonably assumed to be non-dissolvable^43^:

10 $${(\frac{{M}_{t}}{{M}_{\infty }})}^{2}=kt$$

The zero-order release model is commonly used to describe the dissolution behaviours of modified-release pharmaceutical matrices and may also be used to describe the drug release from composite nanoparticles^44^. The mathematical model is given by

11 $$\frac{{M}_{t}}{{M}_{\infty }}=kt$$

Because of the escape of loaded drug molecules, drug-loaded biocompatible sol-gel-processed silica nanoparticles have been demonstrated to be decomposable into smaller fragments^45^. In this case, the dimensions of drug-loaded silica xerogel matrices are not constant during the dissolution of drug molecules. To describe this release behaviour, the following cube-root Hixson-Crowell model^46^ was considered:

12 $$\sqrt[3]{(1-\frac{{M}_{t}}{{M}_{\infty }})}=-\,kt$$

The prepared composite nanoparticles exhibited spherical morphologies. To better describe the drug release behaviours from these spherical diffusion-rate-limiting matrices, the following Baker-Lonsdale model developed from the Higuchi model^47^ was considered:

13 $$\frac{3}{2}[1-{(1-\frac{{M}_{t}}{{M}_{{\infty }}})}^{\frac{2}{3}}]-\frac{{M}_{t}}{{M}_{\infty }}=kt$$

On the basis of the structure of the composite nanoparticles, the drug release was assumed to exhibit a two-stage profile. Stage 1 is characterized by an initial fast release, which reflects the dissolution and diffusion behaviours of the small fraction of drug molecules incorporated into the polymer shells and deposited onto the surface of the silica xerogel core^28^. Drug release in the stage 1 largely relies on the erosion of the polymer shell, which was therefore assumed to exhibit first-order release kinetics. Stage 2 is characterized by a sustained slow release, which describes the combinational effects of the erosion of the polymeric shells as well as the dissolution and diffusion of the drug molecules from the silica xerogel cores. Because of the porous structure of the spherical silica xerogel as well as the porous structure of the eroded polymeric shell, the stage 2 was assumed to be best described by the Baker-Lonsdale model. As most of the drug would be released in stage 2, the drug release of both stages might be best described by the Baker-Lonsdale model. To confirm these assumptions, all of the aforementioned release models were applied to describe experimental drug release profiles.

**Figure 6:**
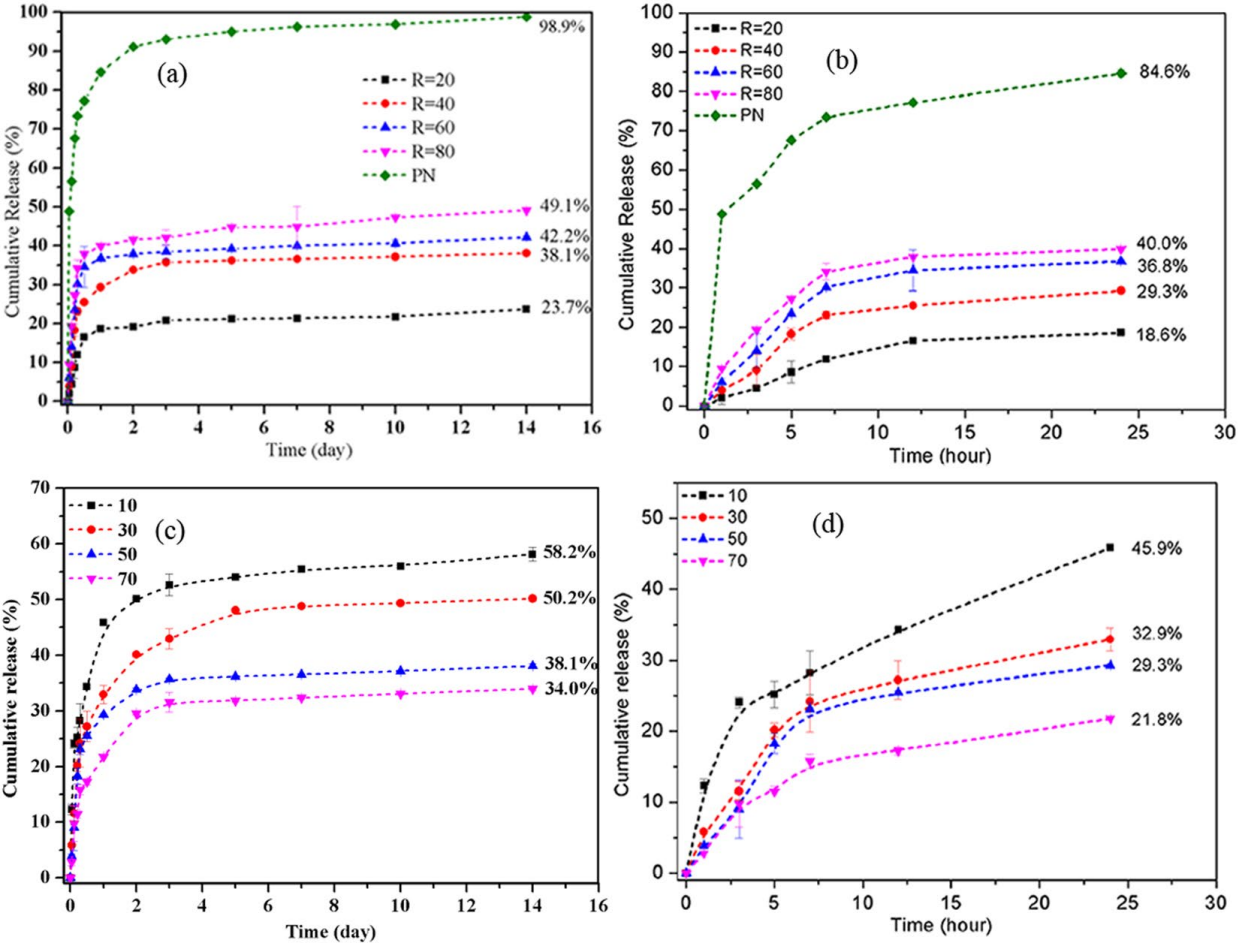
Cumulative drug releases of different formulations. (**a**) Full release profiles and (**b**) release profiles (<24 hours) of composite nanoparticles with different *R*_*W/T*_ ratios and conventional polymer nanoparticles (PN); (**c**) Full release profiles and (**d**) release profiles (<24 hours) of composite nanoparticles prepared with different polymer concentration and a constant *R*_*W/T*_ ratio of 40.

On the basis of the theoretical analysis, control of the drug release kinetics was achieved by adjusting the polymer shell and the silica xerogel core. To confirm this assumption, the cumulative drug release of the composite nanoparticles fabricated with different *R* ratios and different polymer concentrations was measured. In this study, the drugs encapsulated in the nanoparticles were assumed to be the total releasable drug amount in the experiment. Definition of the encapsulated drug is stated in the section of “determination of drug loading and encapsulation efficiency”. The comparisons of drug release are based on the drug released fraction of total releasable amount of each sample. Compared with the conventional polymer nanoparticles, the composite nanoparticles exhibit a much slower release rate as shown in Fig. 6(a,b) due to the presence of the silica xerogel. Generally, the drug release from the composite nanoparticles over 14 days (336 h) was controlled from 23.7% to 49.1% by adjusting the *R*_*W/T*_ ratio from 20 to 80. This control was possible because increasing the *R*_*W/T*_ ratio contributed to a high porosity and large pore size of the silica xerogel matrix and, thus, a relatively high release rate. The cumulative drug release over 14 days was controlled in the range from 34.0 to 58.2% by the adjustment of the polymer concentration from 10 to 70 mg/ml at a constant *R*_*W/T*_ ratio of 40. As shown in Fig. 6(c,d), faster drug release was observed at a lower polymer concentration because the drug molecules penetrated through the thinner polymeric shell more rapidly. By manipulating the polymer concentration, the drug release over 1 day could be varied from 18.6% to 84.6%, whereas the drug release over 14 days could be tailored from 23.7% to 100%.

To confirm the aforementioned assumption of a two-stage release profile, some analyses corresponding to both single-stage release and two-stage release were carried out by applying the proposed drug release equations for the drug release curves of each formulation. According to the single-stage analysis results (Tables S3 and S4), the Baker-Lonsdale model exhibited the best fit among the mathematical models for the drug release kinetics considered in the present study. Although the Baker-Lonsdale model is a derivative of the Higuchi model, the *R* value of the Baker-Lonsdale model was higher than that of the Higuchi model, indicating that the particle shape influenced the drug release behaviour of the nanoparticles. For the conventional polymer nanoparticles, the first-order kinetics model exhibited the best fit, which demonstrates that in the absence of silica xerogel cores, drug release of the polymer nanoparticles was primarily determined by the erosion behaviour of the polymer shells.

Regression analyses of the release profiles with different mathematical models were also conducted on a two-stage basis. For the two-stage analysis, the release profile of each stage was analysed independently with mathematical models. As indicated in Fig. 6(a,c), the first release stage is the release profile within 24 h, whereas the second release stage is the release profile after 24 h, as judged on the basis of the significant differences between the slopes of the release curves. Similar to the previous finding, the Baker-Lonsdale model also exhibited the best fit among all of the studied models for each stage. Modelling of the two-stage basis resulted in better-fitting results (Tables 1 and 2) than modelling of the single-stage release curves, as shown by comparison with the correlation coefficient (*R*) values listed in Tables S3 and S4, suggesting that analysis on a two-stage basis is a better route for interpreting the drug release behaviours of the composite nanoparticles.

**Table 1:** Correlation coefficient (*R*) for fitting the release profiles of composite nanoparticles with different *R* ratios on a two-stage basis.

Model/equation	*R*_*W/T*_ = 20		*R*_*W/T*_ = 40		*R*_*W/T*_ = 60		*R*_*W/T*_ = 80	
	Stage 1	Stage 2	Stage 1	Stage 2	Stage 1	Stage 2	Stage 1	Stage 2
Zero Order	0.906	0.929	0.850	0.913	0.831	0.995	0.803	0.982
First Order	0.915	0.931	0.867	0.917	0.853	0.996	0.831	0.985
Hixson and Crowell	0.912	0.931	0.861	0.916	0.846	0.996	0.822	0.984
Baker and Lonsdale	0.959	0.935	0.932	0.924	0.908	0.996	0.892	0.987
Higuchi	0.957	0.935	0.925	0.921	0.899	0.996	0.882	0.985

**Table 2:** Correlation coefficient (*R*) for fitting the release profiles of composite nanoparticles with different polymer concentration on a two-stage basis.

Model/equation	10 mg/ml		30 mg/ml		50 mg/ml		70 mg/ml	
	Stage 1	Stage 2	Stage 1	Stage 2	Stage 1	Stage 2	Stage 1	Stage 2
Zero Order	0.888	0.949	0.870	0.888	0.850	0.913	0.877	0.917
First Order	0.931	0.957	0.894	0.896	0.867	0.917	0.893	0.922
Hixson and Crowell	0.918	0.955	0.886	0.893	0.861	0.916	0.886	0.920
Baker and Lonsdale	0.993	0.960	0.963	0.903	0.932	0.924	0.972	0.929
Higuchi	0.988	0.956	0.955	0.898	0.925	0.921	0.968	0.926

The *k* values (release rate constants) were used to quantify and compare the drug release profiles of different formulations. The *k* value of each formulation was determined by applying the Baker-Lonsdale model (Tables S5 and S6). The release rate constants of the composite nanoparticles are much smaller than that of the conventional polymer nanoparticles, which confirms the effectiveness of the composite nanoparticles for long-term drug release applications. The release rate (*k* value) is also proportional to the *R*_*W/T*_ ratio and inversely proportional to the polymer concentration for both release stages, confirming the previous assumption that the *R*_*W/T*_ ratio and polymer concentration were the two basic factors controlling the drug release rate. A regression analysis was also performed to better illustrate the relationships between the *k* values and *R*_*W/T*_ ratio or polymer concentrations. Linear relationships are observed for the *k* value of stage 1 for both groups of formulations (Figs S3 and 4). A second-order polynomial relationship between the *k* value and *R* ratio was obtained; it indicates that increasing the *R*_*W/T*_ ratio contributes to a substantial increase of the drug release rate (Fig. S3(b)). A significant reduction of the *k* value was also observed between the polymer concentrations of 30 and 50 mg/ml (Fig. S4(b)). Changes of the *k* values were mild at a polymer concentration less than 30 mg/ml or greater than 50 mg/ml. This phenomenon is explained by the fact that a much better drug encapsulation was achieved at polymer concentrations equal to or greater than 50 mg/ml. In summary, the drug release rate could be precisely manipulated by adjusting the *R*_*W/T*_ ratio and/or the polymer concentration according to their relationships with the *k* values.

In all, the drug release kinetics of the composite nanoparticles could be effectively manipulated by changing the compositions of the polymer shells and core materials, in terms of the *R*_*W/T*_ ratio and polymer concentration. To lower the release rate, the strategy of increasing the polymer concentration or decreasing the *R*_*W/T*_ ratio, as observed in this study, can be adopted. The slowest drug release would be achieved by selecting the highest polymer concentration and the lowest *R*_*W/T*_ ratio simultaneously. To achieve a slower initial release and a relatively fast continuous release rates, the selection of a high polymer concentration and a high *R*_*W/T*_ ratio would be a proper strategy because the sustained drug release rate (stage 2) is primarily dictated by the dissolution and diffusion of drug molecules from the silica xerogel cores, especially during the later stage of the erosion of polymer shells.

## Methods

PLGA (Mw ≈ 30 kDa, PLA/PGA ratio: 50/50) and PLGA-PEG (Mw ≈ 11 kDa) were acquired from the Jinan Daigang Biomaterials Co., Ltd (Jinan City, China). Dichloromethane (DCM) and HCl and NH_3_ solutions were obtained from the Merck & Co. Polyvinyl alcohol (Mw ≈ 23 kDa) and tetraethyl orthosilicate (TEOS) were purchased from the Sigma-Aldrich. Vancomycin hydrochloride was obtained from the Amresco.

### Fabrication of composite nanoparticles

Based on the gelation emulsion method developed in our previous work^28^, minor modifications were further made to the primary emulsion process to improve the stability of the primary emulsion for the gelation reaction. Briefly, TEOS, H_2_O and HCl were mixed with a molar ratio of 1:20:0.0002 under mild agitation at room temperature until TEOS was completely hydrolysed to give a clear silica solution. A calculated amount of vancomycin was dissolved in 0.6 ml of the 0.15–2.4% NH_3_ solution. Next, 0.4 ml of the prepared silica solution was ultrasonically homogenized using an ultrasonic cell crusher (model SKL250-11N, Ningbo Haishu Sklon Electronic Instrument Co. Ltd.) into 10 ml of polymer solution (shell material) to form the primary emulsion. The polymer solution was prepared by dissolving a predetermined amount of PLGA and PEG-PLGA with a weight ratio of 3:2 into 10 ml of DCM. Then, 0.6 ml of NH_3_/vancomycin solution was added gradually during the emulsification of the primary emulsion to initiate the gelation of the silica solution. Afterward, this primary emulsion was dispersed into 22 ml of 0.5% PVA aqueous solution to generate a secondary emulsion under sonication for a predetermined time. A suspension of vancomycin-loaded silica xerogel/polymer composite nanoparticles was obtained after 3 h of mild agitation for solvent evaporation. The nanoparticle suspension was centrifuged at 12,000 rpm using a high-speed centrifuge (model TG16-W, Hunan Xiangyi Centrifuge Instrument Co., Ltd.) for 30 min, and the supernatants were kept for drug concentration analysis using UV/vis spectrophotometry (model UV1102, Techcomp Ltd.). Conventional PLGA nanoparticles were prepared using the same process parameters as the composite nanoparticles except that the silica solution and NH_3_ solution were replaced with pure water.

### Characterization

The morphology and structure of the samples were observed by scanning electron microscopy (SEM) (JEOL JSM-6335F). Compositional information of the composite nanoparticles was obtained by Fourier transform Infrared infrared (FTIR) spectroscopy (Thermo Scientific Nicolet IS50). The average size of the nanoparticles was determined by a Zetasizer Nano ZS (Malvern Instruments, Malvern, U.K.) instrument.

### Determination of drug loading and encapsulation efficiency

The concentration of vancomycin in the supernatant was determined by measuring its absorbance via UV spectrophotometry at 280.5 nm. A standard curve, which was used to calibrate the relationship between the drug concentration and UV absorbance, was prepared using drug concentrations ranging from 0.35 to 0.0024 mg/ml in 0.5% of PVA. The standard curve of drug concentration in response to UV absorbance was determined to be y = 0.16672 × −0.00421, with a correlation factor *R*-square of 0.999 in regression analysis. The encapsulation efficiency of the vancomycin in the nanoparticles was determined by identifying the concentration of the non-encapsulated free drug in the supernatant after centrifugation of the nanoparticle suspension at 12,000 rpm for 30 min. The encapsulation efficiency of the drug-loaded nanoparticles was calculated using the following equation:

$${Encapsulation}\,{Efficiency}\,( \% )=\tfrac{{Total}\,{drug}\,{amount}-{Free}\,{drug}\,{amount}}{{Total}\,{drug}\,{amount}}\times 100 \% $$

The drug loading capacity is defined as follows:

$${Drug}\,{loading}\,{capacity}\,( \% )=\tfrac{{Total}\,{encapsulated}\,{drug}\,{amount}}{{Total}\,{polymer}\,{amount}}\times 100 \% $$

### *In vitro* drug release

An appropriate amount of nanoparticles containing 3 mg of vancomycin was dispersed into 40 ml of phosphate buffer solution (PBS) (pH = 7.2 ± 0.2) in a 50-ml tube with a cap. The tube was shaken at 100 cycles/min in a gas bath shaker with a constant temperature of 37.0 ± 0.2 °C. Then, 4 ml of dissolution medium was withdrawn from each tube at a fixed time interval. After centrifugation at 12,000 rpm for 10 min, 2 ml of supernatant from each of the collected dissolution medium samples was filtered through a 0.22 μm membrane before analysis of the drug concentration via UV/vis spectrophotometry at 280.5 nm. All the dissolution media were added back to their corresponding tubes after the analysis was completed.

## Electronic supplementary material

Supplementary information
