# Supplementary material for: Effects of Compositional Tailoring on Drug Delivery Behaviours of Silica Xerogel/Polymer Core-shell Composite Nanoparticles
Source: Sci Rep. 2018 Aug 29;8:13002. doi: 10.1038/s41598-018-31070-9 (PMC6115385; doi:10.1038/s41598-018-31070-9)
Supplement: Supplementary file 1 — Supplementary information [file 41598_2018_31070_MOESM1_ESM.docx]

**Supporting Information**

**Effects of Compositional Tailoring on Drug Delivery Behaviors of
Silica Xerogel/Polymer Core-shell Composite Nanoparticles**

**^1^Wenfei Huang, *^1^Chi Pong Tsui, ^1^Chak Yin Tang, ^2^Linxia Gu**

^1^Department of Industrial and Systems Engineering, The Hong Kong Polytechnic University, Hung Hom, Kowloon, Hong Kong, China

^2^Department of Mechanical and Materials Engineering, University of Nebraska-Lincoln, NE 68588-0656, USA

^*Corresponding author: E-mail address:^ [^mfgary@polyu.edu.hk^](mailto:mfgary@polyu.edu.hk)

**Content**





**Figure S1** Particle size distribution of composite nanoparticles (average size =301.8nm)


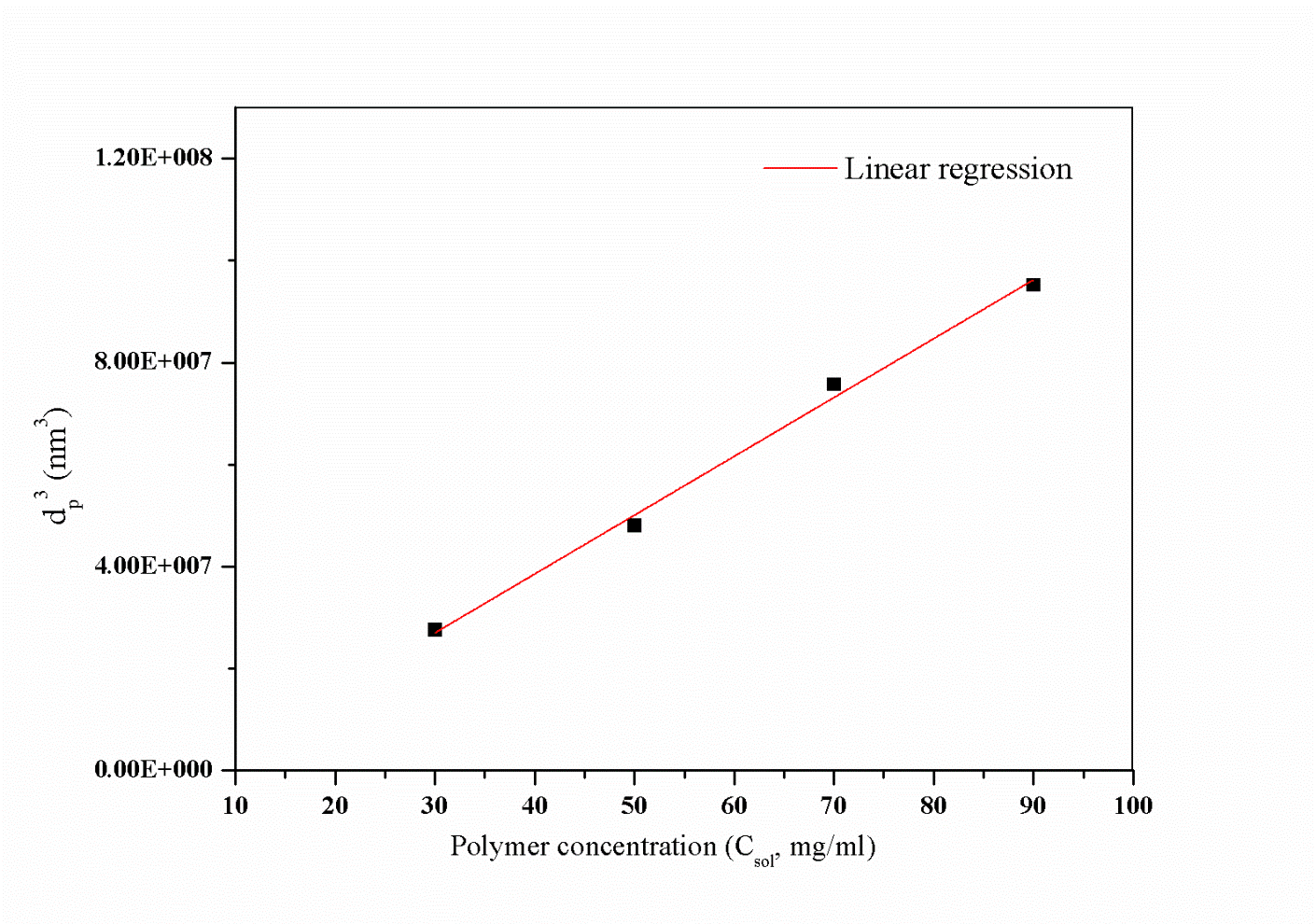

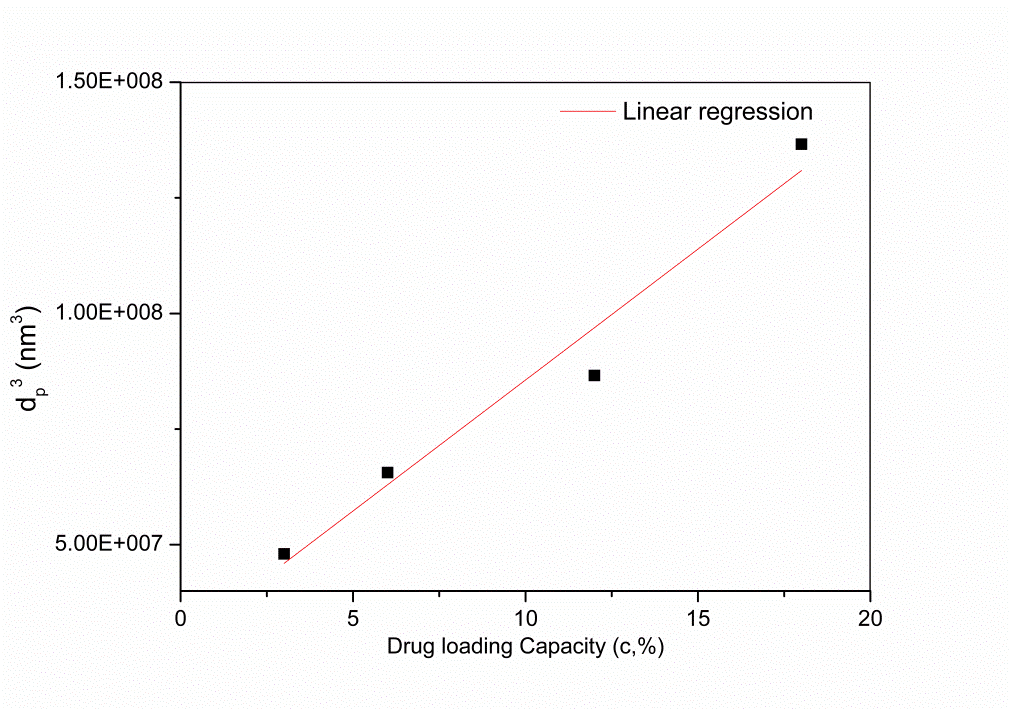


(a)

(b)

**Figure S2** (a) Relationship between the diameter of composite nanoparticle (*d_p_^3^*) and polymer concentration (Csol); (b) Relationship between the drug loading capacity (C) and cubic diameter (*d_p_^3^*)


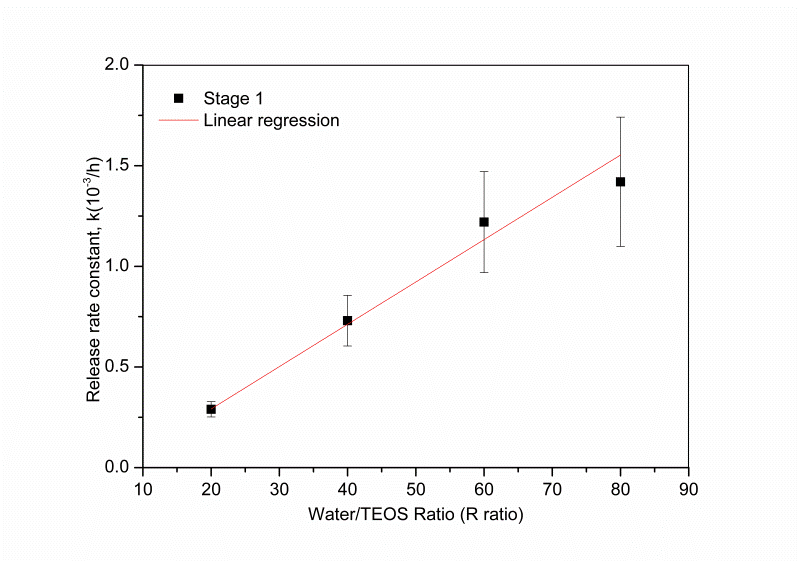

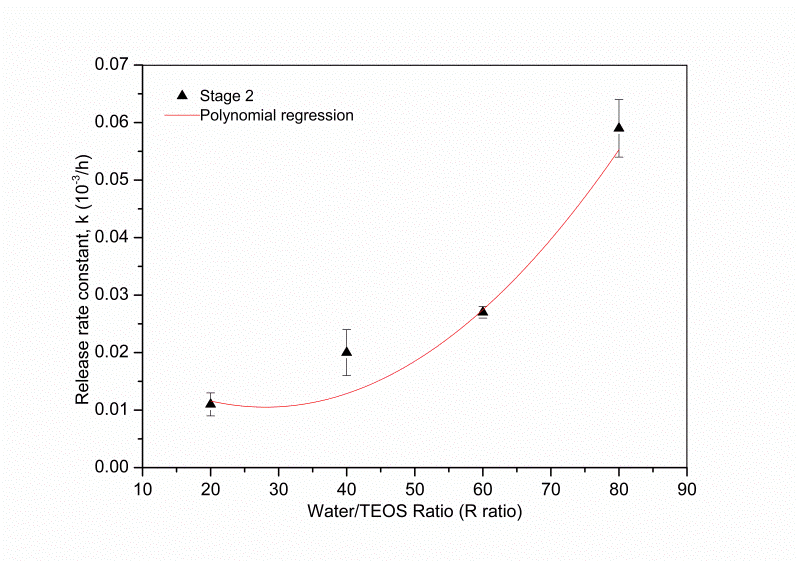


(a)

(b)

**Figure S3** Release rate constants, *k*, and the corresponding regressions of (a) stage 1 and (b) stage 2 for composite nanoparticles of different *R_W/T_* ratios


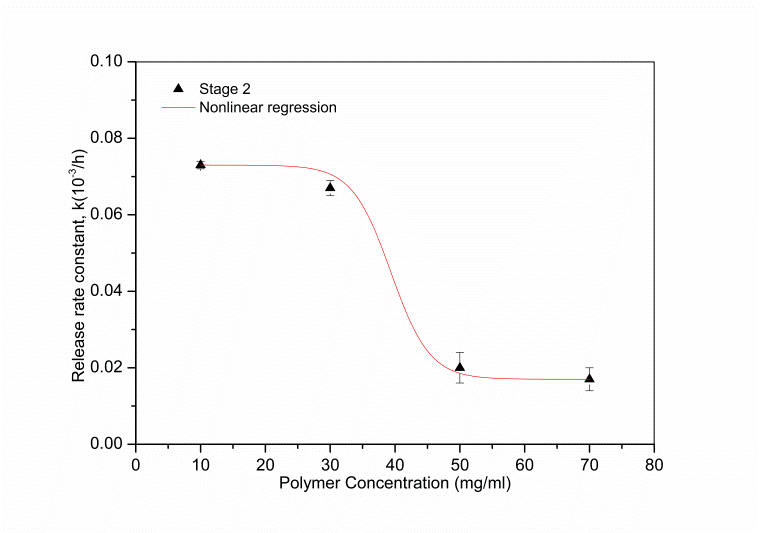

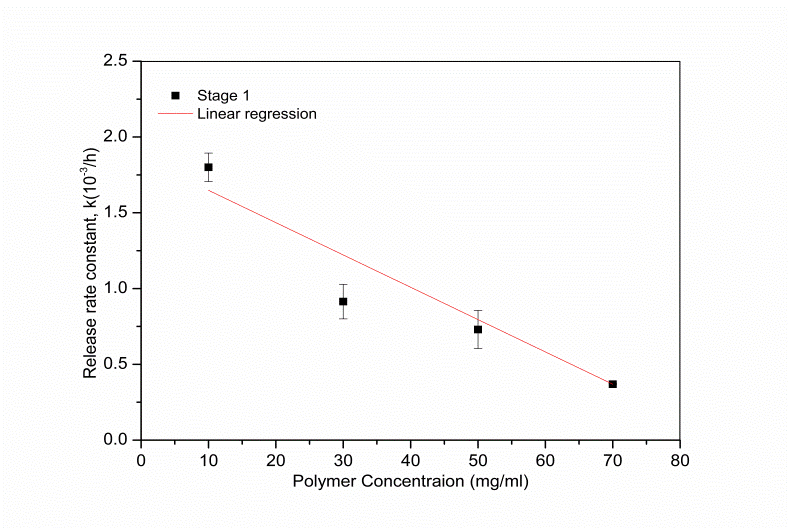


(a)

(b)

**Figure S4** Release rate constant, k, and the corresponding regressions of (a) stage 1 and (b) stage 2 for composite nanoparticles of different polymer concentration

**Tables**

|  | Value | Standard error | *P* value |
| --- | --- | --- | --- |
| Intercept | -7.61×10^6^ | 3.51×10^6^ | 0.16 |
| Slope | 1.15×10^6^ | 0.055×10^6^ | 0.002 |

**Table S1** The regression parameters of the linear relationship between *ρ_sol_* and *d_p_^3^*

|  | Value | Standard error | *P* value |
| --- | --- | --- | --- |
| Intercept | 29.00×10^6^ | 8.53×10^6^ | 0.077 |
| Slope | 5.66×10^6^ | 0.75×10^6^ | 0.017 |

**Table S2** The regression parameters of the linear relationship between *C (%)* and *d_p_^3^*

| Model | *R_W/T_* =20 | *R_W/T_* =40 | *R_W/T_* =60 | *R_W/T_* =80 | PN |
| --- | --- | --- | --- | --- | --- |
| Zero Order | 0.476 | 0.672 | 0.604 | 0.643 | 0.587 |
| First Order | 0.499 | 0.703 | 0.640 | 0.697 | 0.905 |
| Hixson and Crowell | 0.491 | 0.693 | 0.628 | 0.679 | 0.807 |
| Baker and Lonsdale | 0.640 | 0.783 | 0.722 | 0.793 | 0.846 |
| Higuchi | 0.626 | 0.770 | 0.703 | 0.766 | 0.719 |

**Table S3** Correlation coefficient (*R*) for fitting release profiles of conventional polymer nanoparticles (PN) and composite nanoparticles with different *R* ratios on a single-stage basis

| Model | 10mg/ml | 30mg/ml | 50mg/ml | 70mg/ml |
| --- | --- | --- | --- | --- |
| Zero Order | 0.711 | 0.753 | 0.672 | 0.740 |
| First Order | 0.765 | 0.796 | 0.703 | 0.762 |
| Hixson and Crowell | 0.748 | 0.782 | 0.693 | 0.755 |
| Baker and Lonsdale | 0.824 | 0.860 | 0.783 | 0.825 |
| Higuchi | 0.802 | 0.844 | 0.770 | 0.817 |

**Table S4** Correlation coefficient (*R*) for fitting release profiles of composite nanoparticles with different polymer concentrations on a single-stage basis

| Model | Dissolution rate constant, *k*, (10^-3^/h) | | | |  |
| --- | --- | --- | --- | --- | --- |
|  | *R_W/T_* =20 | *R_W/T_* =40 | *R_W/T_* =60 | *R_W/T_* =80 | PN |
| Stage 1 | 0.290 (± 0.038) | 0.73 (± 0.126) | 1.22(± 0.251) | 1.42(± 0.321) | 8.28(±1.63) |
| Stage 2 | 0.011 (± 0.002) | 0.020(± 0.004) | 0.027(± 0.001) | 0.059 (± 0.005) | 0.462(±0.044) |

**Table S5** Release rate constants, *k*, of drug releases for conventional polymer nanoparticles (PN) and composite nanoparticles with different *R_W/T_* ratios (calculated by Baker and Lonsdale model)

| Model | Dissolution rate constant, *k*, (10^-3^/h) | | | |
| --- | --- | --- | --- | --- |
|  | 10mg/ml | 30mg/ml | 50mg/ml | 70mg/ml |
| Stage 1 | 1.8 (± 0.094) | 0.914 (± 0.0.114) | 0.73 (± 0.126) | 0.369 (± 0.004) |
| Stage 2 | 0.073 (± 0.001) | 0.067(± 0.020) | 0.020(± 0.004) | 0.017 (± 0.003) |

**Table S6** Release rate constants, *k*, of drug releases of composite nanoparticles with different polymer concentrations (calculated by Baker and Lonsdale model)
